# Epithelial-myoepithelial carcinoma of the nasopharynx: cases report and literature review

**DOI:** 10.3389/fonc.2025.1720988

**Published:** 2026-01-16

**Authors:** Wenju Xiong, Lijuan Yin, NaNa Wu, Zhongzheng Xiang, Jun Wang, Yuanyuan Zeng, Xiaoyuan Wei, Zelei Dai, Chenfeng Tan, Lei Liu

**Affiliations:** 1 Department of Head and Neck Oncology, Cancer Center & State Key Laboratory of Biotherapy, West China Hospital, Sichuan University, Chengdu, China; 2 Department of Pathology, West China Hospital, Sichuan University, Chengdu, Sichuan, China; 3 Department of Oncology, Chengdu First People’s Hospital, Chengdu, Sichuan, China

**Keywords:** ^18^F-FDG, case report, comprehensive treatment, epithelial-myoepithelial carcinoma, lung metastasis, nasopharynx

## Abstract

**Background:**

Epithelial-myoepithelial carcinoma (EMCa) is a low-grade malignant tumor that occurs primarily in the salivary glands. Its occurrence in the nasopharynx is exceedingly rare. Due to its scarcity, there is currently a lack of standardized treatment protocols, and robust prognostic data remain limited.

**Methods:**

We report a case series of two patients with histopathologically confirmed nasopharyngeal EMCa, supplemented by a systematic literature review to summarize its clinicopathological characteristics, treatment modalities, and prognostic factors.

**Results:**

The two cases exhibited strikingly divergent outcomes. The first patient (Case 1) underwent surgical resection followed by adjuvant radiotherapy, achieving a complete clinical response and remaining disease-free at 31 months. In contrast, the second patient (Case 2) presented a markedly more aggressive profile, characterized by younger age, neurological symptoms, and an atypically high ^18^F-FDG uptake on PET/CT, a notable deviation from the typically non-significant avidity reported for EMCa. Despite aggressive multimodal therapy, the disease progressed rapidly with extensive metastases. Subsequent institution of a combined chemotherapy and immunotherapy regimen successfully achieved disease stabilization.

**Conclusion:**

Our cases illustrate the heterogeneous clinical behavior of nasopharyngeal EMCa, ranging from indolent disease to aggressive progression. Our observations suggest that high ^18^F-FDG uptake may serve as a critical biomarker for an aggressive phenotype. Given this variability, individualized treatment strategies may be warranted, and further studies are needed to better define optimal management for this rare tumor.

## Introduction

Epithelial-myoepithelial carcinoma (EMCa), accounting for approximately 1-2% of all salivary gland neoplasms (1–4), was initially reported by Donath et al. in 1972 (5) and was classified as a low-grade malignancy for the first time in 1991 by the World Health Organization (WHO) (6). Histopathologically, EMCa is characterized by biphasic tubular structures composed of an inner layer of ductal epithelial cells and an outer layer of clear myoepithelial cells (7). EMCa most commonly arises in the major salivary glands, particularly the parotid and submandibular glands (8, 9). It has also been reported at multiple anatomical sites, including regions that contain minor salivary glands, most commonly the palate (10), as well as in the pituitary gland (11), lacrimal gland (12–18), maxillary sinus (19, 20), nasopharynx (21–23), tongue base (24–26), subglottic region (27), trachea (28–30), lung (31–33), bronchial (34–36), pleura mediastinal (37), breast (38–41), kidney (42) and penile region (43). Among these locations, primary involvement of the nasopharynx is particularly rare, with only a limited number of cases reported to date (21–23). Due to the low incidence of EMCa, standardized treatment guidelines have not been established. Radical surgical resection continues to be the mainstay of therapeutic management, while the evidence supporting the benefit of adjuvant radiotherapy remains limited. Most knowledge about EMCa comes from cases originating in the salivary glands. In contrast, primary EMCa of the nasopharynx is rare, and the nasopharynx is deeply situated and closely associated with the skull base, cranial nerves, and major vessels, making complete surgical resection challenging. As a result, the treatment of nasopharyngeal EMCa differs from that of EMCa arising in the salivary glands. Management of nasopharyngeal EMCa typically involves a multimodal approach, including surgery, radiotherapy, and systemic therapy. Given its rarity and challenging location, primary nasopharyngeal EMCa warrants further study to better understand its behavior and optimize treatment. Here, we report two additional cases of female patients with nasopharyngeal EMCa and review the relevant literature. We aim to enhance understanding and knowledge of this disease. We present the following cases in accordance with the CARE reporting checklist.

## Case report

### Case 1

**Figure 1:**
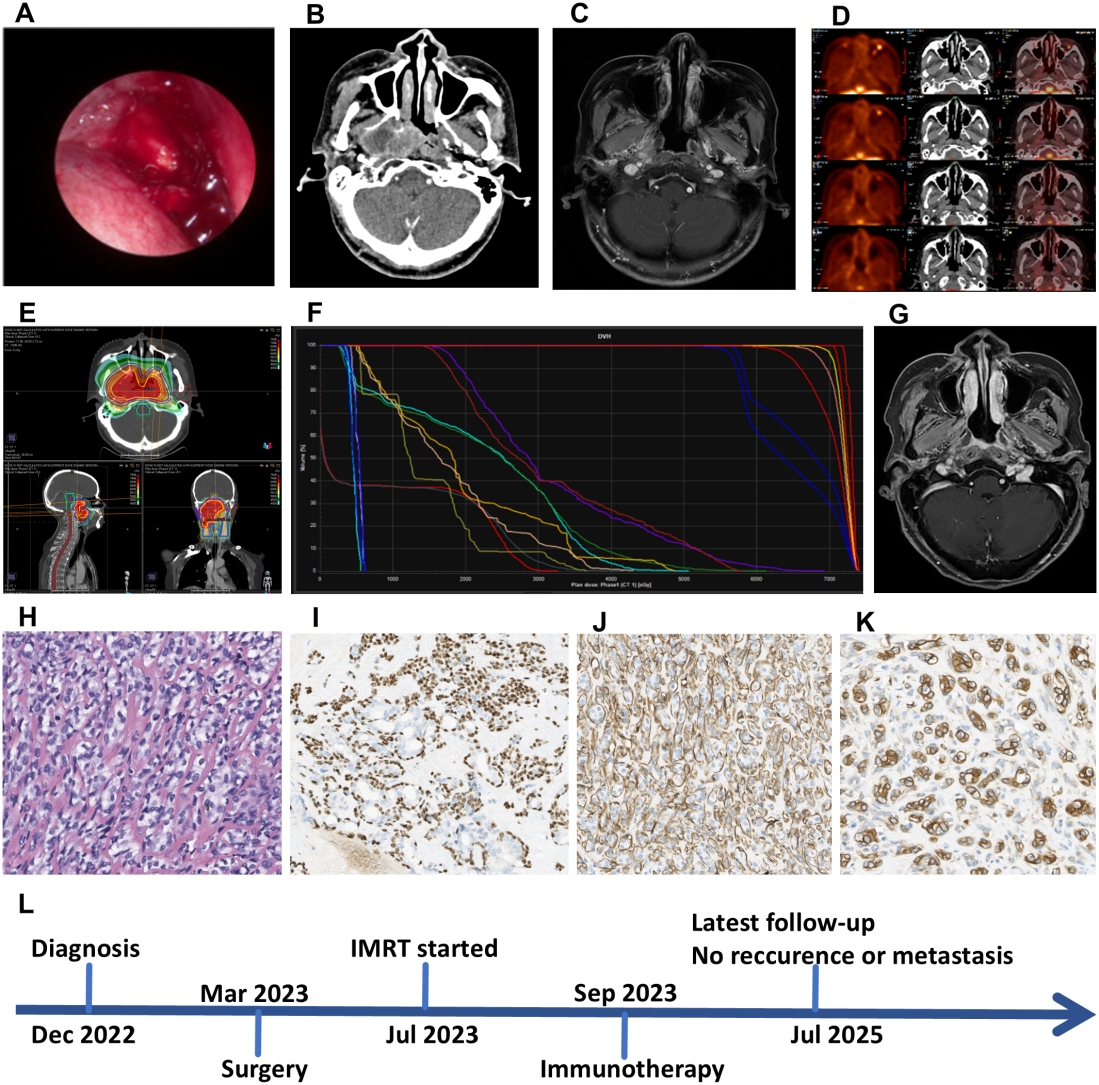
**(A)** Endoscopic view of the nasopharynx showed a friable, bulging mass occupying the right nasopharyngeal space. **(B)** Axial contrast-enhanced CT scan revealed a heterogeneously enhancing mass in the right lateral nasopharyngeal wall, associated with destruction of the pterygoid plate. **(C)** Postoperative axial contrast-enhanced T1-weighted MRI showed mild linear enhancement in the right nasopharyngeal surgical bed, a nonspecific finding that could represent postoperative change. **(D)** Axial fused ^18^F-FDG PET/CT image demonstrated mild ^18^F-FDG uptake (SUV_max_, 2.34) in the nasopharynx, which was interpreted as postoperative inflammation rather than viable tumor. **(E)** Radiotherapy dose distribution. Axial, sagittal, and coronal CT images showed the 95% isodose line (red) conformally covering the planning target volume (PTV) while sparing organs at risk (OARs). **(F)** Dose-volume histogram (DVH). The DVH curves provided a quantitative summary of the radiation dose delivered to the PTVs and critical OARs. **(G)** Follow-up axial contrast-enhanced T1-weighted MRI showed no evidence of an enhancing soft tissue mass to suggest tumor recurrence. **(H)** EMCa H&E staining (magnification, x40), fibrous tissue separating cell clusters. Immunohistochemical staining showed positive staining for p63 **(I)** (magnification, x40), SMA **(J)** (magnification, x40), while CK7 was negative **(K)** (magnification, x40). **(L)** Imaging and treatment timeline of the patient.

A 65-year-old female with no history of chronic diseases was hospitalized with a history of foreign body sensation in the pharynx, occasionally accompanied by snoring. In December 2022, nasal endoscopic examination revealed a soft mass in the nasopharynx, which partially bulged into the right nasal cavity (**Figure 1A**). Enhanced CT showed a 4.6 × 2.7 × 4.5 cm bulging mass with heterogeneous enhancement on the lateral wall of the nasopharynx, along with destruction of the pterygoid plate (**Figure 1B**).

#### Treatment and outcome

Navigation-guided endoscopic resection of the skull base tumor with nasopharyngectomy was performed on March 30, 2023. Three months postoperatively, axial contrast-enhanced T1-weighted MRI showed mild enhancement of the surgical area (**Figure 1C**). On June 28, 2023, a PET/CT scan revealed increased FDG uptake in the nasopharyngeal and oropharyngeal regions, with an ^18^F-FDG maximum standardized uptake value (SUV_max_) of 2.34 (**Figure 1D**). This finding was attributed to postoperative inflammation. Considering the risk of tumor recurrence, the patient underwent intensity-modulated radiotherapy (IMRT) with a total dose of 6996 cGy delivered in 33 fractions (from July 24 to September 7, 2023) (**Figures 1E, F**). The patient subsequently received maintenance immunotherapy with tislelizumab for a period of one year. As of the latest follow-up on July 11, 2025, there was no evidence of local recurrence or distant metastases (**Figure 1G**). According to the RECIST 1.1 criteria, the patient had achieved a clinical complete response (cCR). Both overall survival (OS) and progression-free survival (PFS) were 31 months from the time of initial diagnosis in December 2022.

#### Histopathological findings

Under the microscope, the tumor exhibits a predominant arrangement of transparent muscle epithelial cells, forming solid nests of clear cells with intervening fibrous tissue showing glassy degeneration. A small number of ductal epithelial cells are present within the nests. Hematoxylin and eosin (H & E) staining reveals a locally spindle dual-phase structure, representing the typical histological characteristics of EMCa (**Figure 1H**). Immunohistochemistry demonstrates positivity for P63 in the outer layer of transparent cells, with nuclear expression (**Figure 1I**), as well as positivity for smooth muscle actin (SMA) in the cytoplasm (**Figure 1J**), consistent with a myoepithelial phenotype. In contrast, the inner layer of cells is positive for cytokeratin 7 (CK7), a marker for epithelial cells (**Figure 1K**), supporting the diagnosis of EMCa.

A timeline summarizing the key clinical events, from initial diagnosis to the last follow-up, is presented in **Figure 1L**.

#### Patient perspective

The patient expressed significant relief following the resolution of the initial pharyngeal foreign body sensation and snoring. She reported high satisfaction with her current quality of life and appreciated the minimal invasiveness of the endoscopic surgery. The patient also expressed gratitude for the clear communication and support from the healthcare team throughout her treatment course.

### Case 2

**Figure 2:**
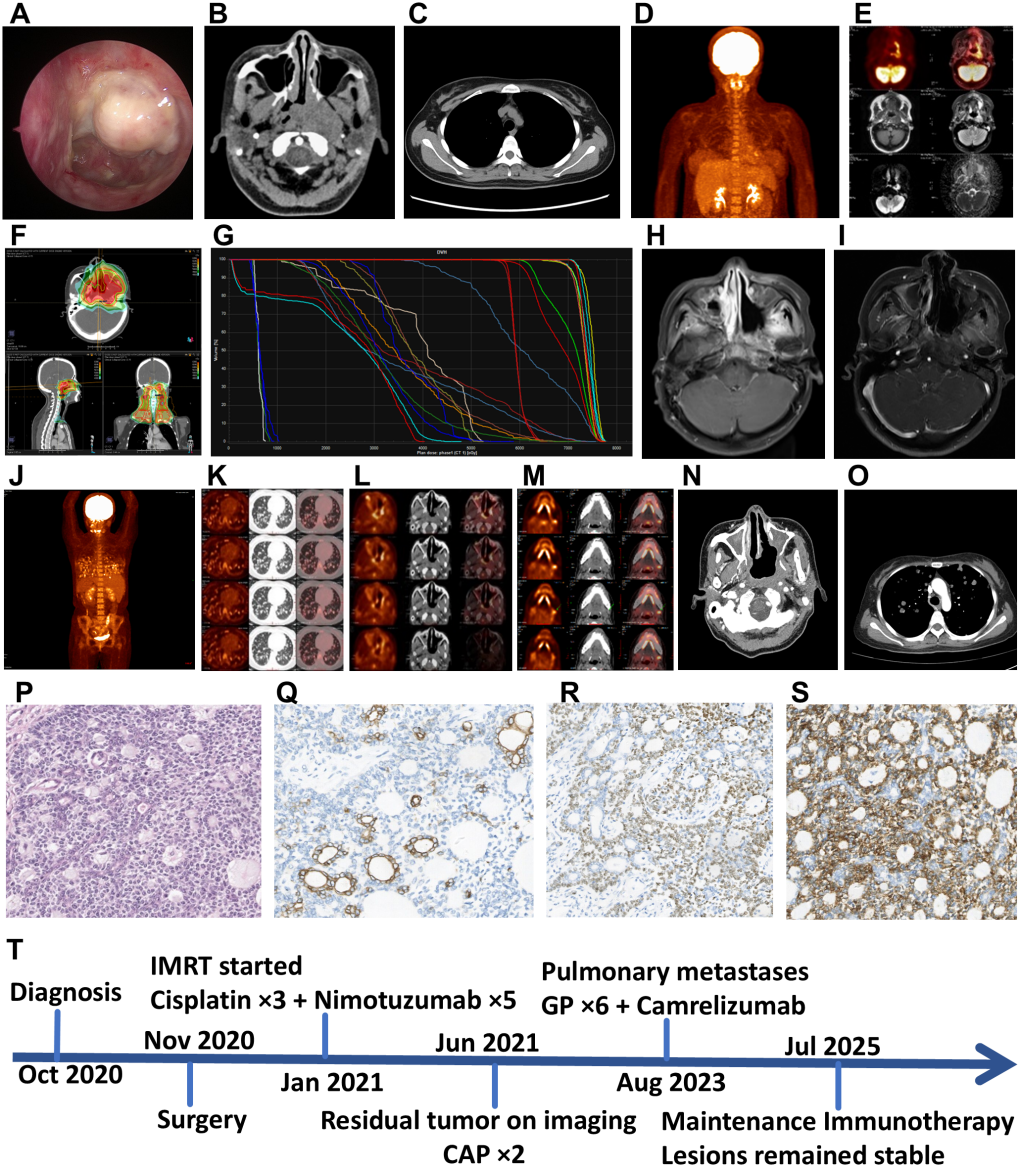
**(A)** Endoscopic view of the nasopharynx showed a bulging mass covered with a pseudomembrane on the left side. **(B)** Axial contrast-enhanced CT scan revealed a mass in the left nasopharyngeal wall, leading to obliteration of the pharyngeal recess and erosion of the nearby bony structures. **(C)** Preoperative chest CT (lung window) showed no signs of pulmonary metastases. **(D, E)** Postoperative ^18^F-FDG PET/MRI: **(D)** The fused PET/MRI image displayed focal ^18^F-FDG avidity (SUV_max_, 6.75) in the left nasopharynx. **(E)** The fused PET/MRI image localized the metabolic activity to a residual soft tissue mass. No distant metastases are observed. **(F, G)** Radiotherapy treatment plan: **(F)** Dose distribution on axial, sagittal, and coronal CT images demonstrated the 95% isodose line (red) conformally covering the PTV while sparing critical organs at risk (OARs). **(G)** The corresponding DVH provided a quantitative analysis of the dose delivered to the PTVs and OARs. **(H)** Axial contrast-enhanced T1-weighted MRI after concurrent chemoradiotherapy showed a persistently enhancing lesion in the left nasopharynx, suggesting residual disease. **(I)** Axial contrast-enhanced T1-weighted MRI after two cycles of adjuvant CAP chemotherapy indicated stable disease with minimal change in the enhancing lesion. **(J-M)**^18^F-FDG PET/CT findings during disease progression: **(J)** The maximum intensity projection (MIP) image showed multiple ^18^F-FDG-avid lesions throughout the body. **(K)** Axial ^18^F-FDG PET/CT image of the chest revealed ^18^F-FDG-avid pulmonary nodules (SUV_max_, 6.84). **(L)** Axial PET/CT image of the head and neck displayed intense ^18^F-FDG uptake (SUV_max,_ 17.73) in the recurrent nasopharyngeal mass. **(M)** Axial ^18^F-FDG PET/CT image confirmed ^18^F-FDG-avid metastasis in a right cervical lymph node (SUV_max_, 7.19). **(N)** Axial contrast-enhanced CT scan of the nasopharynx showed tumor recurrence and extensive bone destruction of the central skull base. **(O)** Follow-up chest CT after systemic therapy indicated stable pulmonary metastatic disease. **(P)** EMCa HE staining (magnification, x40). Immunohistochemistry showed positive staining for Pan-CK **(Q)** (magnification, x40), p63 **(R)** (magnification, x40), and SMA **(S)** (magnification, x40). **(T)** Imaging and treatment timeline of the patient.

A 34-year-old female presented with a 4-year history of progressive unilateral nasal symptoms, including obstruction and epistaxis. Subsequently, the patient developed signs of cranial nerve involvement, including facial numbness, diplopia, and tinnitus. On September 24, 2020, nasal endoscopic examination showed a bulge on the left side of the nasopharynx with a thick pseudomembrane on the surface (**Figure 2A**). CT scans also revealed a 4.5 × 3.0 × 5.0 cm mass in the nasopharynx, leading to obliteration of the left pharyngeal recess and the erosion of the left temporal bone, tubal torus, and foramen lacerum (**Figure 2B**). No conclusive evidence of lung metastases was identified preoperatively (**Figure 2C**).

#### Treatment and outcome

On November 5, 2020, under general anesthesia, the patient underwent navigation-guided endoscopic resection via a combined Caldwell-Luc approach for the removal of a naso-cranial base tumor, unilateral nasopharyngeal tumor, and a maxillary sinus/pterygopalatine fossa tumor, with concurrent left posterior choana plasty and left trigeminal and vidian neurectomies. Postoperative ^18^F-FDG-PET/MRI showed increased uptake in the residual lesions of the left nasopharynx and nasal cavity (SUV_max_, 6.75), accompanied by involvement of the left parapharyngeal space and cavernous sinus. The increased uptake corresponded to a tissue signal on the fused MRI, indicating significant metabolic activity. No evidence of locoregional or distant metastasis was found (**Figures 2D, E**). Due to the presence of residual tumor, the patient underwent cisplatin-based (75 mg/m^2^ intravenously every 3 weeks for up to 3 cycles) concurrent chemoradiotherapy (CCRT), with a total dose of 6996 cGy in 33 fractions (212 cGy/fraction, 5 fractions/week) (**Figures 2F, G**). The patient also received concurrent targeted therapy with nimotuzumab at a dose of 100 mg administered weekly throughout the course of CCRT from January 21 to March 11, 2021. The patient’s pain while swallowing obstruction was significantly relieved. Approximately two months after completing CCRT, in May 2021, the patient reported progressive symptoms indicative of local recurrence, including worsening unilateral nasal obstruction, recurrent epistaxis, left-sided facial hypesthesia, and a persistent deep-seated headache.

In June 2021, a follow-up MRI demonstrated a left-sided nasopharyngeal mass with invasion of the parapharyngeal space and the skull base (left foramen lacerum, clivus, and petrous apex), as well as circumferential encasement of the left internal carotid artery within the carotid canal. The tumor extended anteriorly into the left nasal cavity, with destruction of the middle and inferior turbinates and the medial wall of the left maxillary sinus, and superiorly to the floor of the middle cranial fossa (**Figure 2H**). Considering local recurrence, two cycles of postoperative adjuvant chemotherapy based on the CAP (Cyclophosphamide + Cisplatin + Liposomal Doxorubicin) regimen were administered, with the last session completed in August 2021. The tumor size remained stable, and regular follow-up examinations showed no significant progression (**Figure 2I**).

Nevertheless, the patient complained of retrosternal pain triggered by coughing three years later. In August 2023, a PET/CT scan revealed diffuse solid nodules in both lungs (maximum diameter, 2.0 cm), with intense ^18^F-FDG uptake (SUV_max_, 6.84) (**Figures 2J, K**). Moreover, the scan exhibited a 1.4 × 1.6 × 0.8 cm soft tissue mass on the left wall of the nasopharynx, involving the nasal septum, left inferior nasal meatus, left greater wing of the sphenoid, and pterygoid process with intense ^18^F-FDG uptake (SUV_max_, 17.73) (**Figure 2L**). Furthermore, elevated ^18^F-FDG uptake was observed in most cervical lymph nodes (SUV_max_, 7.19) (**Figure 2M**). According to the RECIST 1.1 criteria, these findings collectively indicated Progressive Disease (PD).

Considering the tumor progression, the patient was treated with a GP regimen (Gemcitabine + Cisplatin) plus Camrelizumab, with response assessments every two cycles. After 6 cycles of combination therapy, the patient was transitioned to maintenance immunotherapy with Camrelizumab alone, achieving sustained disease stabilization. The most recent Head and Chest CT (July 2025) revealed stable disease (SD) per RECIST 1.1 criteria (**Figures 2N, O**).

#### Histopathological findings

In October 2020, the histopathological results of the specimen from an excisional biopsy supported the diagnosis of nasopharyngeal EMCa. A small number of ductal epithelial cells are present within the nests. H & E staining reveals locally biphasic tubular structures, showing the typical histological characteristics of EMCa (**Figure 2P**). Immunohistochemistry results indicated positive staining for pan-cytokeratin (Pan-CK) (**Figure 2Q**), partial positivity for P63 (**Figure 2R**), and positivity for smooth muscle actin (SMA) (**Figure 2S**), confirming the diagnosis of EMCa.

A timeline summarizing the key clinical events, from initial diagnosis to the last follow-up, is presented in **Figure 2T**.

#### Patient perspective

The patient described the profound physical and emotional challenge of dealing with an aggressive cancer at a young age. She highlighted the distress caused by neurological symptoms like facial numbness and diplopia. Despite the toll of multimodal therapy, she conveyed a strong determination to fight the disease. The patient reported that the disease stabilization achieved with immunotherapy and chemotherapy brought her renewed hope and improved her ability to manage daily life.

## Discussion

EMCa is one of several low-grade malignant tumors of the salivary glands. It is generally associated with a relatively favorable prognosis, with reported 5- and 10-year survival rates of 72.7–91.3% and 59.5–90.2%, respectively. However, despite its low-grade classification, EMCa carries a substantial risk of local recurrence, reported in 23–50% of cases (1, 2, 44), and distant metastases may occur in a small number of patients (4.5%-10%) (2, 3, 26, 45). Facial nerve symptoms are rare in EMCa patients, and poorly differentiated types may result in nerve paralysis (4, 7, 45, 46). The average onset age for the occurrence of EMCa is over 60 years old, with a female predominance (57.3%-62.2%). However, our second patient is much younger than the mean age reported in previous literature (2, 3, 45), and accompanied by a series of neurological invasion symptoms. Despite aggressive antitumor therapy, metastases developed in both lungs and regional lymph nodes. The rapid progression in this patient may be linked to the tumor’s location in the nasopharynx. Tumors in this area lie close to the skull base, cranial nerves, and major blood vessels, which makes surgery challenging and limits the ability to achieve clear margins. Planning radiotherapy is also difficult because of these critical structures. These factors likely contributed to the local progression seen in Case 2. Typically, EMCa has been known to have a non-significant ^18^F-FDG uptake on PET/CT, which is often associated with its low malignant potential (32, 47, 48). Interestingly, in the case of the second patient, we observed an atypical high uptake of ^18^F-FDG (SUV_max_, 17.73) in the lesions. Our findings are consistent with Kamga’s (49). We summarized the clinical and ^18^F-FDG uptake characteristics of reported cases of EMCa with lung metastases (26, 50–58) (**Table 1**). In all cases of EMCa with lung metastases, only two case reports have described similar multiple metastatic lesions in both lungs with mild-to-moderate FDG uptake (58, 59). We propose that the atypical presentation on PET scan of pulmonary metastases might be associated with the tumor’s malignant degree, which reflects the significant metabolic activity and Poor prognosis. Hence, imaging appearances are non-specific for EMCa. Histological and immunohistochemical studies can achieve a more accurate diagnosis of the disease.

**Table 1:** Clinical and imaging characteristics of the cases of epithelial−myoepithelial carcinoma with lung metastases based on the literature review.

Case/Year	Age/Sex	Primary sites	Initial treatment	Interval to metastasis	Metastatic sites	^18^F-FDG uptake	Metastasis therapy	Outcome
Noel et al, 1992 (50)	63/M	Left parotid gland	Surgery	14 years after surgery	Both lungs	NA	NA	Minimal tumor growth 5 months after lung metastases
Kasper et al, 1999 (51)	58/F	Left parotid gland	Surgery	12 years after surgery	Right lung and Bones	NA	Palliative Chemotherapy with 5-FU	Died 15 years after initial diagnosis
Pierard et al, 2006 (52)	51/M	Submandibular gland	Surgery and Adjuvant radiotherapy	3 years after surgery	Both Lungs and Brain	NA	Chemotherapy + Radiotherapy (Cisplatin + 5-FU → Paclitaxel → cyclophosphamide + Pan-encephalic radiotherapy)	Died 30 months after initial diagnosis
Yang et al, 2012 (53)	60/F	Left Submandibular gland	Surgery	15 months after surgery	Both Lungs	NA	Refused	Lost to follow up
Yamazaki et al, 2013 (54)	35/M	parotid gland	Surgery and Adjuvant radiotherapy	10 months after surgery	Both Lungs	NA	Chemotherapy (TPF regimen)	No apparent recurrence or metastasis 2 years after chemotherapy
Hsieh et al, 2016 (55)	43/M	Right parotid gland	Surgery and Adjuvant radiotherapy	3 years after the appearance of previous stable lung nodules	Left Lung	NA	video-assisted thoracoscopic wedge resection	Died 30 months after initial diagnosis
Chen et al, 2017 (26)	52/M	Base of tongue	NA	Discovered together with the primary lesion	Both Lungs	no significant FDG uptake	Palliative care service	Died 18 months after initial diagnosis
Mäkelä et al, 2020 (56)	36/F	Salivary gland	Surgery	4 years after surgery	Right Lung	NA	First-line chemotherapy (cisplatin, docetaxel, and 5- FU), targeted therapy (Everolimus, and Trastuzumab emtansine plus Lapatinib), palliative care	Died shortly after stopping of active treatment
Civan et al, 2024 (57)	51/F	Parotid gland	Surgery	5 years after surgery	Both Lungs	Mild to moderate FDG uptake	Chemotherapy (capecitabine plus cisplatin)	NA
Wang et al, 2025 (58)	60/M	left parotid gland	Surgery	5 years after surgery	Both Lungs	Mild to moderate FDG uptake	Refused	No recurrence or abnormally enlarged lung nodules 6 years after surgery and 20 months after lung metastases

F, female; M, male; CK, cytokeratin; PCK, pan-cytokeratin; CK7, cytokeratin 7, SMA, smooth muscle actin; 5-FU, 5-Fluorouracil; PD, Progressive Disease per RECIST 1.1 criteria; CR, Complete Response per RECIST 1.1 criteria; SD, Stable Disease per RECIST 1.1 criteria.

On microscopic examination, typical sections of EMCa show nests, tubules, and gland-like structures composed of epithelial and myoepithelial cells. However, other malignant salivary gland tumors exhibit this biphasic differentiation pattern, including adenoid cystic carcinoma (ADCC) and basal cell adenocarcinoma (BCAC); hence, the diagnostic criteria for EMCa should include eosinophilic ductal cells and hyaline myoepithelial cells (7). The infiltrative growth and various cell ratios lead to different histologic subtypes. Some common subtypes include a sieve-like growth pattern, papillary-cystic pattern, basal-like appearance, and sebaceous differentiation, accounting for about 17%-18% of all EMCa cases (7, 52). Since the varieties of EMCa in H&E staining, immunohistochemical features contribute to a more accurate diagnosis, the epithelial cells of EMCa show intense positive staining for markers, such as CK, CK7, the myoepithelial cells of EMCa typically exhibit strong positivity for markers, such as SMA, calmodulin, p63, and S-100, among which, p63 is the most sensitive and specific one (2, 7, 21–23). Studies have found that the codon 61 (Q61) of the Harvey murine sarvey rat sarcoma viral oncogene homolog (HRAS) is present in 65% of EMCa patients. EMCa is often associated with HRAS mutations (82.7%), and the sites are mostly located in codon 61 of exon 3 (HRAS Q61R) (60). DOG1 is expressed in more than 50% of EMCa patients (61), and SOX10 can also be expressed in EMCa as a marker of intercalary tube differentiation (62).

Additionally, we systematically searched databases, including PubMed, Web of Science, Google Scholar, and the search timeframe was from the inception of the databases up to September 10, 2025, and identified three previously reported cases of nasopharyngeal EMCa (21–23). Together with the two patients we report here, a total of five nasopharyngeal EMCa cases were included. The baseline clinical and immunohistological characteristics are summarized in **Table 2**. The mean age at diagnosis of EMCa was 58 years, with a female predominance (80%). Treatment was classified according to the initial therapeutic approach. Three patients (60%) underwent surgical resection as the primary treatment, all followed by postoperative adjuvant (chemo)radiotherapy. The remaining two patients (40%) received definitive primary (chemo)radiotherapy. During follow-up, two patients suffered from locoregional recurrence: one from the surgery group and one from the primary chemoradiotherapy group. Furthermore, distant metastasis was observed in one patient (20%) from the surgical group, despite having undergone resection and multimodal adjuvant therapy. All patients were confirmed to be alive at the end of their respective follow-up periods. Among the patients with recurrence, the average age was 42.5 years. Due to the rarity of EMCa, the standard treatment guideline remains unclear. Wide surgical excision followed by optimal adjuvant therapy might offer a mode of treatment with better survival results for EMCa patients. postoperative adjuvant radiotherapy is commonly recommended for patients with aggressive factors, such as tumors larger than 4 cm, those with positive surgical margins, perineural infiltration, lymph node metastases, high-grade status, or vascular infiltration. However, the effectiveness of postoperative adjuvant radiotherapy of EMCa patients remains uncertain (93.2% vs 87.6%, *p=0.483)* (2, 3, 44, 45). Additionally, systemic therapy for recurrent or metastatic EMCa is generally based on standard cytotoxic drugs such as doxorubicin, cisplatin, 5-fluorouracil, paclitaxel, and navelbine (63, 64). Moreover, the application of ex vivo drug screening together with next-generation sequencing to assess targeted treatment strategies can be important for metastatic EMCa patients (56). However, in nasopharyngeal EMCa, anatomical challenges may contribute to rapid local progression, as seen in Case 2. As a result, standard treatment strategies developed for salivary gland EMCa may not be fully applicable in this setting. Nasopharyngeal EMCa often requires a multimodal treatment approach. In the second patient we reported, the addition of immunotherapy brought the disease under control. Therefore, immunotherapy might be an effective treatment strategy for EMCa patients. Nevertheless, these findings are based on limited reports, and more research is needed to fully understand and develop treatment strategies.

**Table 2:** Baseline Clinicopathological characteristics of reported cases of nasopharyngeal epithelial-myoepithelial carcinoma.

Case	Age/Sex	Tumor size	Clinical manifestation	Epithelial markers	Myoepithelial markers	Treatment	Outcome
Imate et al. (21)	60/F	NA	Ear pain	EMA, Ber; EP-4 AE1/AE3, MNF116, S-100a and b antibodies	SMA	Surgery	No recurrence in 55 months
Kim et al. (22)	80/F	2.8×2.6cm	Nose blockage, Epistaxis	CK7	SMA	CCRT + Chemotherapy	No recurrence in 24 months
Zhang et al. (23)	51/M	5.6×3.4cm	Swallowing pain, Ear tinnitus	CK7	P63, SMA, VIM	CCRT	Recurrence 33 months after initial diagnosis
Patient 1	65/F	4.6×2.7x4.5cm	Foreign body sensation, Sleep snoring	CK7	P63, SMA	Surgery + Radiotherapy + Immunotherapy	No recurrence 31 months after surgery
Patient 2	34/F	4.5×3.0x5.0cm	Nasal obstruction, Epistaxis, Hyposmia Hypogeusia, Tinnitus, and Facial numbness, Diplopia, Hearing loss	Pan-CK	P63	multi-line comprehensive anti-tumor therapy	Recurrence and lung metastasis 24 months after CAP regimen treatment. No recurrence or abnormally enlarged lung nodules 23 months after lung metastases.

F, female; M, male; CK, cytokeratin; PCK, pan-cytokeratin; CK7, cytokeratin 7, SMA, smooth muscle actin; CCRT, concurrent chemoradiotherapy; CAP, Cyclophosphamide + Cisplatin + Liposomal Doxorubicin.

## Conclusion

EMCa is generally a low-grade tumor, but it carries a significant risk of local recurrence and metastasis. Standard treatment guidelines remain unclear. Nasopharyngeal EMCa presents additional challenges due to its deep location and proximity to critical structures, which can make surgery difficult and limit local control. Radiotherapy is also constrained, and systemic therapy or immunotherapy may be needed for recurrent or metastatic cases. For these reasons, early radical resection, followed by appropriate adjuvant therapy and careful long-term follow-up, is particularly important for patients with nasopharyngeal EMCa. A multimodal approach is often necessary to improve outcomes and manage this rare tumor effectively.

## Data Availability

The original contributions presented in the study are included in the article/supplementary material. Further inquiries can be directed to the corresponding author.

## Glossary

BCAC: basal cell adenocarcinoma
CAP: Cyclophosphamide + Cisplatin + Liposomal Doxorubicin (regimen)
CCRT: concurrent chemoradiotherapy
cCR: clinical complete response
CK: Cytokeratin
CK7: cytokeratin 7
DVH: dose-volume histogram
EMCa: epithelial-myoepithelial carcinoma
^18^F-FDG: 18F-fluorodeoxyglucose
GP: Gemcitabine + Cisplatin (regimen)
H&E: hematoxylin and eosin
HRAS: Harvey rat sarcoma viral oncogene homolog
IMRT: intensity-modulated radiotherapy
OARs: organs at risk
OS: overall survival
Pan-CK: pan-cytokeratin
PD: Progressive Disease
PET/CT: Positron Emission Tomography/Computed Tomography
PFS: progression-free survival
PTV: planning target volume
RECIST: Response Evaluation Criteria in Solid Tumors
SD: Stable Disease
SMA: smooth muscle actin
SUVmax: maximum standardized uptake value
VIM: vimentin
